# Endothelin Modulates Rhythm Disturbances and Autonomic Responses to Acute Emotional Stress in Rats

**DOI:** 10.3390/biology12111401

**Published:** 2023-11-05

**Authors:** Eleni-Taxiarchia Mouchtouri, Thomas Konstantinou, Panagiotis Lekkas, Alexandra Lianopoulou, Zoi Kotsaridou, Iordanis Mourouzis, Constantinos Pantos, Theofilos M. Kolettis

**Affiliations:** 1 Department of Cardiology, Medical School, University of Ioannina, 45500 Ioannina, Greece; elenimouch@gmail.com (E.-T.M.); thkon8@gmail.com (T.K.); 2 Cardiovascular Research Institute, 45500 Ioannina, Greece; panlek1981@gmail.com; 3 School of Applied Biology and Biotechnology, Agricultural University of Athens, 10447 Athens, Greece; alexandralianopoulou@gmail.com (A.L.); zoikotsaridou@gmail.com (Z.K.); 4 Department of Pharmacology, National and Kapodistrian University of Athens, 11527 Athens, Greece; imour@med.uoa.gr (I.M.); cpantos@med.uoa.gr (C.P.)

**Keywords:** acute emotional stress, sympathetic activity, vagal activity, endothelin, bradyarrhythmias, tachyarrhythmias

## Abstract

**Simple Summary:**

Our body’s reaction to acute emotional stress differs, depending on several factors, which include a small molecule called endothelin. Excessive responses are important, as they can trigger (sometimes serious) heartbeat disturbances. To further investigate the role of endothelin in this process, we compared the reactions to laboratory-induced stress in two groups of rats, known to have diverse baseline levels of endothelin in the blood. We found distinct responses leading to slow heartbeat disturbances in the latter group. This feature resembles that observed in people with a tendency to faint after strong emotions, a common problem with many unknown aspects. Our study calls for further research on the effects of endothelin on fainting and irregular heartbeat after acute emotional stress.

**Abstract:**

The ubiquitous peptide endothelin is currently under investigation as a modulatory factor of autonomic responses to acute emotional stress. Baseline plasma levels of endothelin alter blood pressure responses, but it remains unclear whether autonomic activity and arrhythmogenesis (i.e., brady- or tachyarrhythmias) are affected. We recorded sympathetic and vagal indices (derived from heart rate variability analysis), rhythm disturbances, voluntary motion, and systolic blood pressure after acute emotional stress in conscious rats with implanted telemetry devices. Two strains were compared, namely wild-type and ET_B_-deficient rats, the latter displaying elevated plasma endothelin. No differences in heart rate or blood pressure were evident, but sympathetic responses were blunted in ET_B_-deficient rats, contrasting prompt activation in wild-type rats. Vagal withdrawal was observed in both strains at the onset of stress, but vagal activity was subsequently restored in ET_B_-deficient rats, accompanied by low voluntary motion during recovery. Reflecting such distinct autonomic patterns, frequent premature ventricular contractions were recorded in wild-type rats, as opposed to sinus pauses in ET_B_-deficient rats. Thus, chronically elevated plasma endothelin levels blunt autonomic responses to acute emotional stress, resulting in vagal dominance and bradyarrhythmias. Our study provides further insights into the pathophysiology of stress-induced tachyarrhythmias and syncope.

## 1. Introduction

Acute emotional stress (AES) is defined as an actual or anticipated threat to well-being [1]. Under conditions of acute threat, the autonomic nervous system alters the physiologic state of many organs, preparing animals or humans to react or retreat, often referred to as the “fight-or-flight” state [2]. These responses are sustained by activation of the hypothalamic–pituitary–adrenocortical axis, leading to elevated circulating glucocorticoids and catecholamines [1,2].

The heart is highly innervated by sympathetic and vagal fibers that continuously control its performance. Acting on the sinus node, the atrioventricular node and the ventricular myocardium, autonomic activity modulates cardiac output to meet the demands associated with the “fight-or-flight” state [3]. However, excessive sympathetic activity enhances automaticity and alters ventricular repolarization, thereby creating an arrhythmogenic milieu in the ventricular myocardium; on the other hand, excessive vagal activity triggers bradycardia which can lead to syncope [4]. Epidemiologic studies [5,6,7] have shown a higher incidence of sudden cardiac death during the days following earthquakes. Such mortality is likely due to rhythm disturbances elicited by shifts in autonomic balance, as shown in experimental studies [8] and clinical case-series reporting anger [9] or fright [10] preceding arrhythmic episodes.

Endothelin (ET)-1 is a ubiquitous 21-amino-acid peptide involved in many biological processes, including the modulation of autonomic activity [11]. Experimental data from several groups [12,13], including ours [14], have demonstrated a complex interplay between the endothelin system and sympathetic activation, which is operative in the adrenal medulla [15], the myocardium [16,17] and the brain [18,19]. Along these lines, rises in ET-1 levels and blood pressure (BP) were found in rats exposed to pulsatile air-jet stress [20], with the vascular endothelium identified as a potential source of ET-1 [21]. In addition to its vascular effects, ET-1 exerts potent actions in the brain and is thought to participate in AES responses, based on its high expression in several loci [22]. Studies in humans have further supported the link between ET-1 and AES, particularly in patient cohorts with chronically elevated ET-1 levels [23,24,25]. A study examining spectators of a sports game reported higher plasma ET-1 levels during excitement in patients with a history of coronary artery disease, accompanied by enhanced sympathetic responses [23]. Furthermore, a small-scale clinical study in patients with atherosclerotic peripheral vascular disease found excessive plasma ET-1 levels after mental stress [24]. Importantly, high endothelin levels have also been associated with vagal dominance in neurocardiogenic syncope, characterized by bradycardia, which is commonly observed after AES [25]. However, current understanding of the modulatory actions of ET-1 on autonomic and cardiac rhythm responses after AES remains incomplete.

The aim of the present work was to further investigate the pathophysiologic role of ET-1 in AES, utilizing an established protocol in conscious rats [26]. The working hypothesis was that autonomic activity and arrhythmogenesis after AES may vary, depending on baseline plasma ET-1 levels. As HR and BP responses provide only an estimate of autonomic balance, we also evaluated sympathetic and vagal activity separately using heart rate variability (HRV) analysis. Two rat strains were compared, namely wild-type Wistar and the previously described Wistar–Imamichi stain (generously provided by Prof. M. Yanagisawa, University of Tsukuba, Japan) [27], which displays 10-fold higher plasma ET-1 levels secondary to impaired clearance [28]. Our comparison included temporal changes in autonomic indices and rhythm disturbances over a prolonged observation period encompassing AES and recovery.

## 2. Materials and Methods

### 2.1. Animal Study Population and Ethics

A total of *n* = 56 rats were studied, of which *n* = 28 (19 ± 1 weeks of age, 405 ± 47 g) were wild-type Wistar and *n* = 28 (20 ± 1 weeks of age, 367 ± 71 g) were ET_B_-deficient Wistar–Imamichi. AES was induced in *n* = 10 rats from each strain, along with a sham procedure in equinumerous animals; in addition, *n* = 8 rats were used from each strain in the BP protocol (described below). To overcome the confounding effects of gender, we included only male rats, given the previously reported gender-related differences in HR responses to AES [29]. Measurements obtained from all animals were used for the analyses, without exclusions. The animals were housed in singles in standard Plexiglas cages, under optimal environmental conditions in terms of temperature (20–22 °C), humidity (70%), and light/dark cycles (12/12 h). Tap water and standard rodent chow were provided ad libitum. All procedures were in accordance with the ARRIVE guidelines [30] and European legislation (2010/63/EU), and the study protocol was approved by the regulatory authorities (Regional Municipality of Attica, approval number: 574219).

### 2.2. Experimental Design

We compared changes over time in HR, voluntary activity, autonomic variables and brady- and tachyarrhythmias between four groups, namely wild-type and ET_B_-deficient rats, in the presence or absence of AES. Time points were taken at baseline, during and after AES or a sham procedure. This part of the study included *n* = 40 rats, in which miniature ECG transmitters (TCA-F40, Transoma, New Brighton, MN, USA) were implanted as previously described [16]; care was taken to avoid motion artifacts by securing both leads to the surrounding tissues. All implantations were performed a minimum of five days prior to the main experiments, allowing recovery from the procedure. The cages containing rats with implanted transmitters were placed on top of telemetry receivers (RCA-1020, Transoma), through which the ECG signal was continuously recorded by the acquisition software (ART, Transoma).

### 2.3. Induction of AES

**Figure 1:**
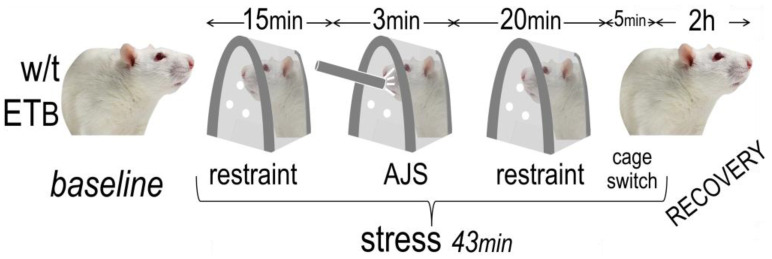
Study protocol. The responses to restraint and air-jet stress (AJS) were examined in wild-type (w/t) (*n* = 10) and ET_B_-deficient rats (*n* = 10); a sham procedure was followed in equinumerous animals.

To account for circadian rhythm as a confounding factor, all experiments were performed during morning hours in a quiet room, under plenty of natural light. We used an established protocol of unpredictable AES, which combines restraint [31] and air-jet stress [32], after slight modifications; the total duration was 43 min, followed by a two-hour recovery period (Figure 1). This protocol is reliably reproduced in the laboratory, with recent validation by our group showing prominent autonomic responses involving both arms [26]. In the sham protocol, the animals remained in their cage and were observed from (a distance of) one meter for the same length of time.

The following five-minute intervals were analyzed: baseline, restraint onset, restrainer-1 (12 min following the onset of the previous period), air-jet (consisting of 18 air-pulses, each of 2 s duration, given at 8 s intervals via air pump at 10 L/min), restrainer-2 (immediately after the termination of the previous period), restrainer-3 (the last five minutes in the restrainer) and exit (the first five minutes in the cage). We included the latter in the period of AES, as it encompasses cage-switch, an established aversive stimulus [33], as reiterated by our recent experience [26]. Findings from the six periods of AES are reported either separately or as their average. In addition, two five-minute periods of recovery were analyzed, namely recovery-1 (commencing five minutes after return to the cage) and recovery-2 (the last five minutes of recovery), reported either separately or as their average. 

### 2.4. Heart Rate Variability Analysis

HRV analysis was performed from consecutive sinus inter-beat intervals, with the widely used, previously validated [34] Kubios software (version 3.3.0, University of Eastern Finland, Kuopio, Finland). In addition to time- and frequency-domain analysis, the software calculates the sympathetic nervous system index (SNSi) and the parasympathetic (vagal) nervous system index (PNSi) by combining several variables; hence, more accurate description of each arm is provided, especially regarding swift changes from steady-state values [34]. The SNSi was computed from three variables, namely (a) mean HR, (b) Baevsky’s stress index, and (c) the length of distribution of Poincaré plots after nonlinear analysis. The PNSi was computed also from three variables, namely (a) the mean inter-beat interval, (b) the root mean square of successive differences between inter-beat intervals in time-domain analysis and (c) the width of the distribution of Poincaré plots. Individual variability in SNSi and PNSi responses was accounted for by their expression as percent change from baseline values.

### 2.5. Arrhythmia Analysis

All stored ECG tracings were analyzed off-line independently by four operators (E-T.M., T K., A.L. and Z.K.), blinded to group identity. Current guides were followed [35], defining premature ventricular contractions (PVCs) as single wide-complex electrical depolarizations interrupting the sinus inter-beat interval; couplets (two consecutive PVCs) and triplets (three consecutive PVCs) were counted accordingly. The number and duration of bradyarrhythmic events were also recorded, including episodes of sinus pause and atrioventricular (AV) block; specifically, sinus pauses were defined as the intermittent absence of atrial and ventricular depolarization, whereas AV block was identified as failing impulse conduction from the atria to the ventricles. The duration of each episode was determined using the graded scale provided by the software (ART, version 2.2., Transoma, New Brighton, MN, USA).

### 2.6. Voluntary Activity

Voluntary activity was recorded with the use of the analysis software (ART, Transoma) at baseline and during the two-hour recovery period. Voluntary activity was depicted as motion counts, totaling the number of changes in animal location within the cage. To account for individual variability, we report the difference between recovery and baseline counts. This variable provides a measure of continuing anxiety and is used as a marker of post-AES adaptation [36].

### 2.7. Blood Pressure Protocol

Systolic BP was recorded noninvasively using the tail-cuff method (IN125/R, ADInstruments, Oxford, UK), as previously described [37]. Because of the restraint required in this method, baseline BP was obtained after acclimatization and prolonged (30 min) stay in the restrainer, as the average of ten successive measurements at the end of this timeframe; subsequently, air-jet pulses were delivered for 3 min, during which two BP measurements were obtained, followed by ten further measurements during a 15 min recovery period. The respective averages were reported as “stress” and “recovery” periods.

### 2.8. Statistical Analysis

Values are reported as mean ± one standard deviation. Baseline variables in both rat strains and voluntary activity post-AES were compared with Student’s *t*-test. Changes over time were assessed with the use of (two-way) analysis of variance for repeated measures, with rat strain and time as between- and within-group factors, respectively. Baseline values of all variables in both ET_B_ groups and both control groups are presented as the respective averages. Differences (between- and within-group) at each prespecified time period were evaluated with the use of the post hoc Tukey’s HSD test. Variables describing brady- and tachyarrhythmias were not normally distributed, according to the (Lilliefors-corrected) Kolmogorov–Smirnov test, and were compared with non-parametric tests, namely Mann–Whitney U-test or Kruskal–Wallis analysis of variance, as appropriate. Statistical significance was set at an alpha level of 0.05.

## 3. Results

### 3.1. Baseline Differences between the Two Rat Strains

Table 1 demonstrates the baseline characteristics in wild-type and ET_B_-deficient rats.

Voluntary activity and HR were comparable between groups, as were time-domain HRV variables. By contrast, the ratio of low- (LF) to high-frequency (HF) spectra in the frequency-domain analysis, depicting steady-state autonomic balance, indicated vagal dominance in ET_B_-deficient rats. This finding reflected differences in both autonomic arms, as shown by lower LF and higher HF values, indicating lower sympathetic and higher vagal activity, respectively.

Lower sympathetic activity in ET_B_-deficient rats was corroborated by lower SNSi, whereas the difference in the vagal index PNSi was of marginal statistical significance. Differences in PVCs or tachyarrhythmias were absent at baseline. However, occasional bradyarrhythmic episodes in the form of sinus pauses were recorded in ET_B_-deficient rats, but not in wild-type rats. No AV conduction disturbances were present.

### 3.2. Autonomic Responses in Wild-Type Rats

**Figure 2:**
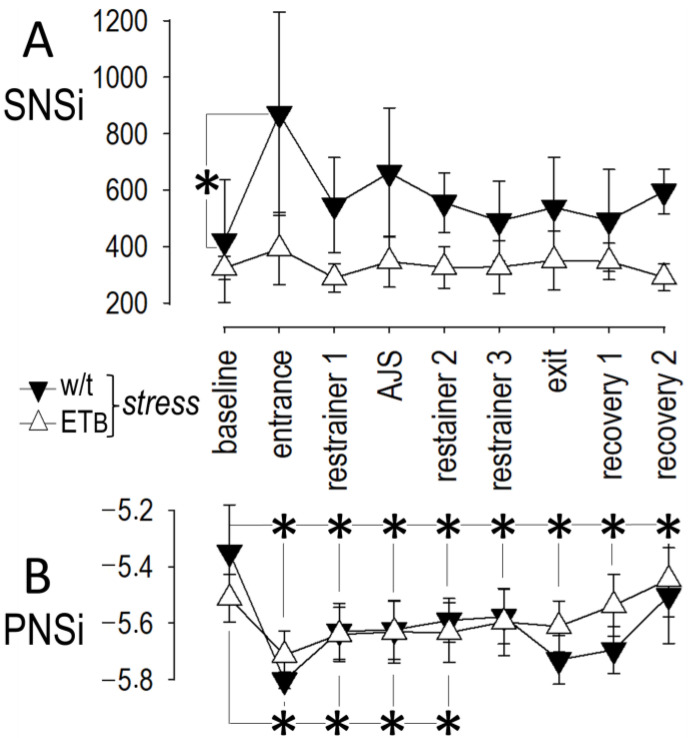
Autonomic responses to acute emotional stress. Detailed responses of (**A**) sympathetic nervous system index (SNSi) and (**B**) parasympathetic (vagal) nervous system index (PNSi) during acute emotional stress and recovery. Asterisks (*) denote significant differences compared to baseline.

Compared to baseline, SNSi increased sharply from baseline in wild-type rats at the onset of AES, with subsequent decline during the remaining period of AES and during recovery (Figure 2A).

Compared to SNSi, changes in PNSi were more prolonged in this rat strain; specifically, PNSi decreased from baseline at the onset of AES and remained low during the period of AES and during recovery (Figure 2B).

### 3.3. Autonomic Responses in ET_B_-Deficient Rats

Contrasting the response observed in wild-type rats, SNSi remained unchanged (from baseline values) during AES and during recovery in ET_B_-deficient rats (Figure 2A). As in wild-type rats, more prolonged PNSi changes were observed in ET_B_-deficient rats; however, PNSi returned to baseline values earlier, i.e., at the end of AES and prior to the onset of recovery (Figure 2B).

### 3.4. Between-Group Comparison: Heart Rate

**Figure 3:**
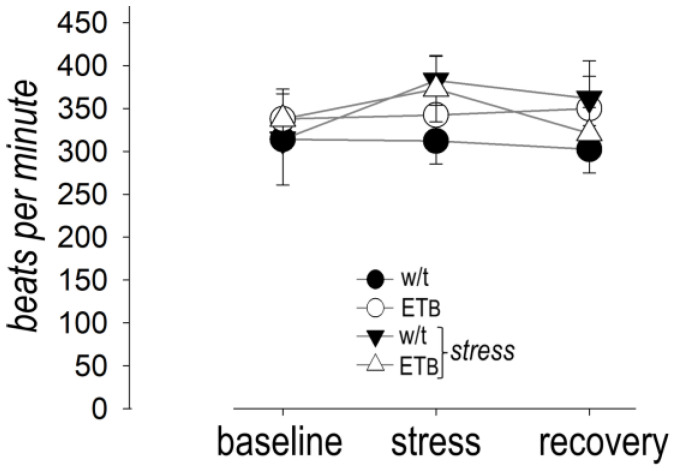
Heart rate. No differences were evident between wild-type (w/t) (*n* = 10) and ET_B_-deficient rats (*n* = 10) during acute emotional stress or recovery.

In the absence of AES, HR remained stable. HR increased during AES and remained high during recovery in wild-type rats. Likewise, HR increased during AES in ET_B_-deficient rats but returned to baseline values during the recovery period. Despite this finding, HR during AES or recovery did not differ significantly between the two rat strains (Figure 3).

### 3.5. Between-Group Comparison: Blood Pressure

**Figure 4:**
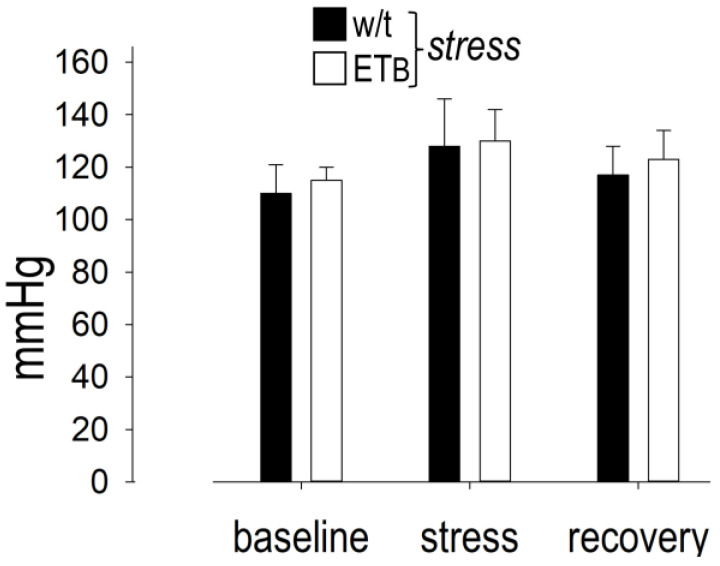
Systolic blood pressure. No differences were evident between wild-type (w/t) (*n* = 10) and ET_B_-deficient rats (*n* = 10) during acute emotional stress or recovery (values expressed in mmHg).

Systolic BP displayed significant differences during AES and recovery in each group, without differences between them (Figure 4). In detail, systolic BP (expressed in mmHg) increased from 110 ± 11 (baseline) to 128 ± 18 during AES and returned to baseline values (117 ± 11) during recovery in wild-type rats. Likewise, systolic BP rose from 115 ± 5 (baseline) to 130 ± 12 during AES and returned to baseline values (123 ± 11) during recovery in ET_B_-deficient rats. The observed differences between the two rat strains failed to reach statistical significance.

### 3.6. Between-Group Comparison: Sympathetic Activity

**Figure 5:**
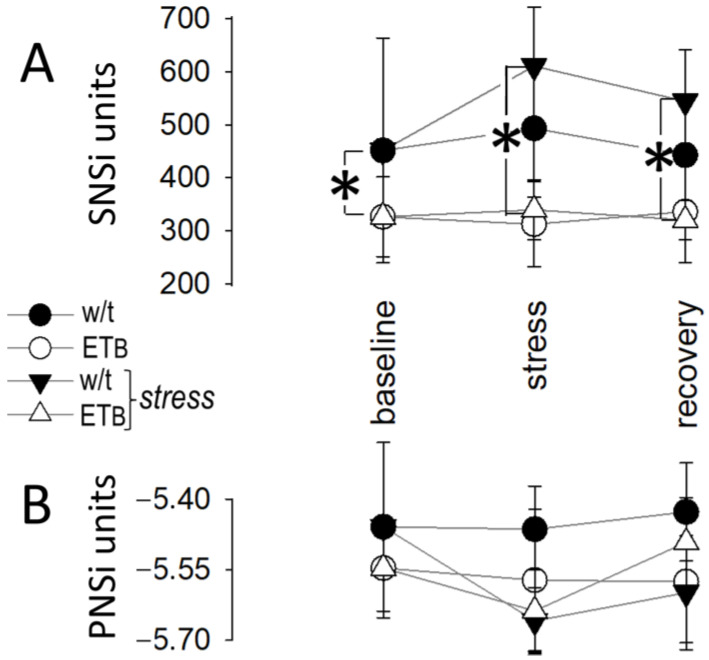
Autonomic activity. (**A**) Sympathetic (SNSi) and (**B**) vagal (PNSi) activity in the four groups (*n* = 10 in each). Asterisks (*) denote significant differences between wild-type (w/t) and ET_B_-deficient rats.

**Figure 6:**
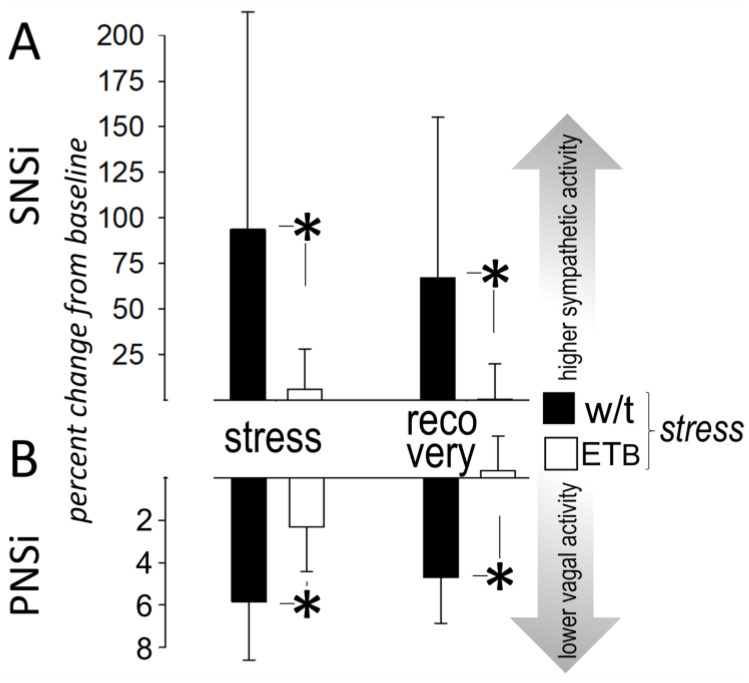
Autonomic responses. Percent changes from baseline in (**A**) sympathetic (SNSi) and (**B**) vagal (PNSi) activity in wild-type (w/t) (*n* = 10) and ET_B_-deficient rats (*n* = 10). Asterisks (*) denote significant differences.

In the absence of AES, sympathetic activity remained stable during the entire observational period in both rat strains, whereas significant differences were observed during AES (Figure 5A). Specifically, sympathetic activation, expressed as SNSi, was markedly higher in wild-type rats than in ET_B_-deficient rats during AES, with this difference persisting during recovery. The higher sympathetic response in wild-type rats was confirmed when SNSi was expressed as percent change from baseline, with major differences observed during AES and during recovery (Figure 6A).

### 3.7. Between-Group Comparison: Vagal Activity

In the absence of AES, vagal activity remained stable during the entire observation period in both groups. During AES, PNSi decreased in both groups, without differences between them (Figure 5B). Despite the more prolonged vagal responses during recovery in wild-type rats (described above, Figure 2B), PNSi did not differ between groups during this timeframe. Importantly, however, PNSi percent changes from baseline were more pronounced in wild-type than in ET_B_-deficient rats during AES and during recovery (Figure 6B).

### 3.8. Between-Group Comparison: Voluntary Motion

**Figure 7:**
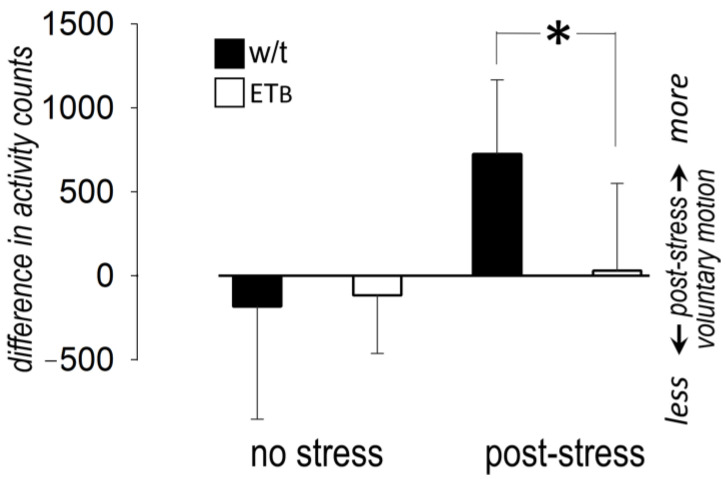
Voluntary motion. Excess voluntary activity (expressed as counts per hour) during recovery (difference from baseline) in the four groups (*n* = 10 in each). Asterisk (*) denotes significant difference between wild-type (w/t) and ET_B_-deficient rats.

In the absence of AES, voluntary motion remained stable during the observational period in both groups. AES had no effect on voluntary motion during recovery in ET_B_-deficient rats, but the difference in activity counts indicated higher motion in wild-type rats (Figure 7).

### 3.9. Between-Group Comparison: Premature Ventricular Contractions

**Figure 8:**
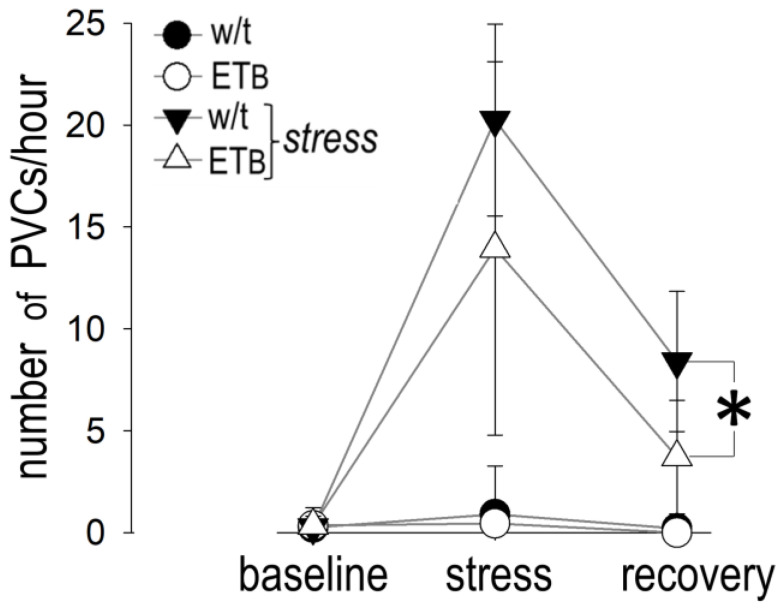
Premature ventricular contractions (PVCs). PVCs were more frequent (asterisk) in wild-type (w/t) (*n* = 10) than in ET_B_-deficient (*n* = 10) rats during recovery.

Figure 8 depicts the number of PVCs per hour at baseline, during AES and during recovery.

During AES, there was a statistical trend (*p* = 0.064), but not significance, towards more frequent PVCs in wild-type rats, when compared to ET_B_-deficient rats. Of note, this difference became significant during the recovery period.

### 3.10. Comparison between Groups: Bradyarrhythmias

**Figure 9:**
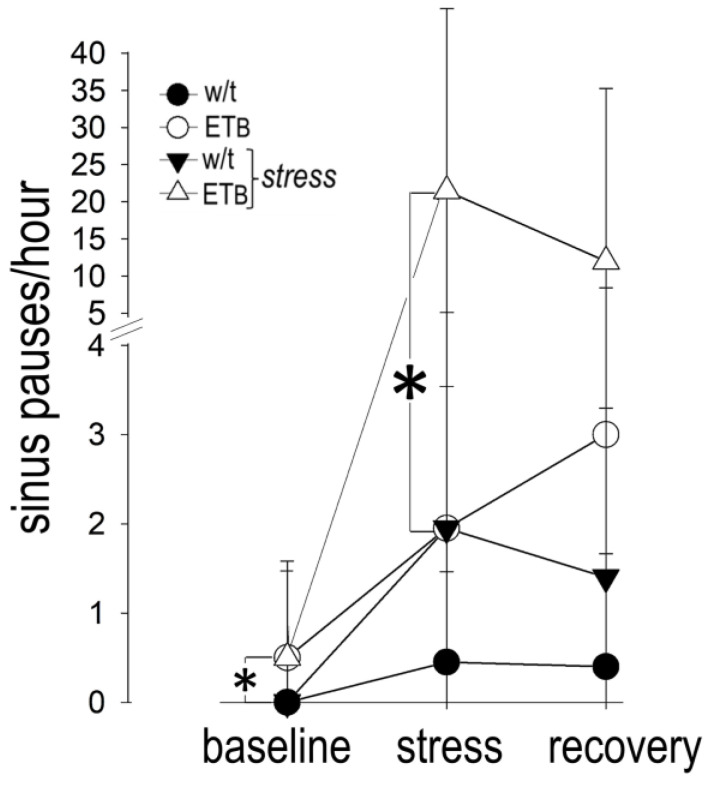
Sinus pauses. More sinus pause episodes (asterisk) were observed in wild-type (w/t) than in ET_B_-deficient rats during acute emotional stress.

Figure 9 depicts the number of sinus pauses during the three periods of observation. 

**Figure 10:**
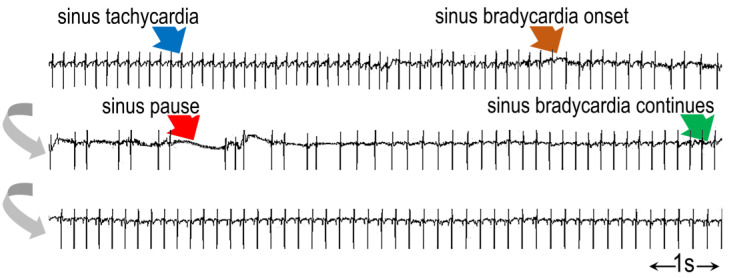
Example of sinus pause in an ET_B_-deficient rat (gray arrows denote continuous strip). Note the sinus tachycardia (~400 bpm, blue arrow) preceding the onset of sinus bradycardia (brown arrow), leading to a sinus pause (red arrow). Sinus bradycardia with discernible P waves resumes (green arrow).

Sinus pauses were more frequent during AES in ET_B_-deficient rats, as compared to wild-type rats. Although sinus pauses were observed in ET_B_-deficient rats during recovery as well, their number per hour was not significantly different from that observed in wild-type rats. Only scarce episodes of AV block were observed during AES or recovery in both groups, without differences between them. An example of a sinus pause is shown in Figure 10. A list of all abbreviations used in the manuscript is given as Supplementary Material (Table S1).

## 4. Discussion

Our experiments demonstrate sympathetic activation and vagal withdrawal in response to AES in wild-type rats. As a result, rises in HR and BP in the range of 20–30% were recorded, which are comparable to those reported in a similar protocol [38]. By contrast, autonomic responses were blunted in ET_B_-deficient rats, especially regarding the sympathetic arm. The markedly different patterns of autonomic activity did not yield differences in HR and BP, but voluntary activity during recovery was higher in wild-type rats, reflecting continuing anxiety in this strain. Moreover, the blunted sympathetic response in ET_B_-deficient rats was accompanied by frequent bradyarrhythmias during AES and recovery, as opposed to frequent PVCs in wild-type rats.

### 4.1. Autonomic Responses in Wild-Type Rats

Despite the widespread use of the rat model of AES, detailed evaluation of sympathetic and vagal responses is scarce. Analyzing time-domain parameters of HRV, Sgoifo et al. [8] described higher sympathetic activation with simultaneous vagal withdrawal during a 15 min recording period after social stress in rats, a model relevant to depressive and anxiety disorders. Complementing our initial experience [26], our current HRV analysis provides an important addition to the characterization of the present AES protocol, which is considered more relevant to emotions of fear [39]. Furthermore, the evolvement of HRV analysis (by combining several variables derived from frequency-domain and nonlinear analysis), as used here, provides a more accurate description of autonomic changes over short periods of time [34].

In our experiments, we observed rapid sympathetic activation at the onset of AES in wild-type rats, with a subsequent plateau at lower values until the end of AES and during recovery. Additionally, vagal withdrawal, which was sustained during the entire observational period, accounted for the continuing anxiety during recovery in wild-type rats. Thus, in keeping with previous findings in rats [40] and humans [41], our results underline the prominent role of vagal withdrawal after AES, participating in the “fight-or-flight” responses to AES [42].

### 4.2. Observational Period Duration

The main aim of the present work was the investigation of the pathophysiologic role of ET-1 in AES. The role of ET-1 in this setting is complex, acting in the brain [43], as well as in the heart [44] and the vascular endothelium [45]. The rapid initial autonomic responses appear to be mediated by the effects of ET-1 on various central loci [38]. ET-1 plasma levels subsequently rise, primarily secreted from the vascular endothelium, an action mediated by corticotropin-releasing hormone [21]. Therefore, our prolonged observation (including 43 min AES and a two-hour recovery period) permitted a comprehensive evaluation of the effects of ET-1. 

### 4.3. Baseline Autonomic Characteristics of ET_B_-Deficient Rats

We utilized the rescued homozygous ET_B_-deficient rat strain, characterized by the absence of functioning ET_B_ receptors in the cardiovascular system. As ET-1 clearance depends on ET_B_ receptors, this rat strain has been a valuable model in various settings, including those investigating the role of chronically elevated plasma ET-1 levels [28]. Our comparison of continuous recordings prior to the induction of AES between wild-type and ET_B_-deficient rats provides further data on this strain. Despite the similar HR and BP, ET_B_-deficient rats had lower sympathetic and higher vagal activity at baseline, reinforcing previous observations of enhancement of vagal reflexes by ET-1, acting either centrally [43] or at peripheral sites of the reflex arc [45]. Moreover, recent findings also indicated lower heart rate as well as impaired baroreflex activity in this strain [46]. Interestingly, occasional sinus pauses were recorded in our ET_B_-deficient but not in wild-type rats, in keeping with the previously reported inhibitory actions of ET-1 on calcium current in the rabbit sinus node [44]. 

### 4.4. Sympathetic Responses in ET_B_-Deficient Rats

The most important finding of the present study was the diverse autonomic pattern after AES in the two rat strains. ET_B_-deficient rats displayed blunted sympathetic response to AES throughout the observation period, a result contrasting the markedly enhanced sympathetic activation previously observed during global [16] or regional [17] myocardial ischemia. This setting is characterized by massive norepinephrine overflow, initially via exocytotic mechanisms, followed by non-exocytotic mechanisms, a process augmented by the effects of ET-1 on ET_A_ receptors [12]. However, the experiments of the present work, investigating the effects of AES on the normal myocardium, are not comparable to the experimental setting of the ischemic myocardium, in which the effects of sympathetic activation differ substantially. 

Our results are in line with those reported in a similar experimental setting, in which high baseline ET-1 levels (induced by means of prior high-salt diet) abrogated the pressor response to air-jet stress in wild-type rats [47]. Of note, diverse BP responses were reported after AES in wild-type and ET_B_-deficient rats in the background of chronic behavioral stress (induced by early-life stress) [48]. In the latter work, wild-type rats had high circulating levels of ET-1 after early-life stress and exhibited enhanced BP response to air-jet stress during adult life; however, this difference was absent in the presence of much higher chronic elevations in plasma ET-1, such as those seen in ET_B_-deficient rats [48]. Previous findings, suggesting decreased functional activity of ET-1 in the presence of chronically elevated plasma levels [28], provide an explanation for the diverse responses in the two strains, although further investigation is required.

### 4.5. Vagal Responses in ET_B_-Deficient Rats

As in wild-type rats, vagal withdrawal was also evident in our ET_B_-deficient rats, but the magnitude of this effect differed, as shown by the percent changes in PNSi. Our findings shed more light on the interaction between ET-1 and vagal activity, a topic that remains poorly understood. In previous work conducted in male Wistar rats, dual (ET_A_ and ET_B_) endothelin receptor blockade for 7 days increased sympathetic drive and lowered vagal activity, likely indicating peripheral sites of action in the reflex arc [45]. In addition to these effects, several pieces of evidence suggest a potent interaction between ET-1 and vagal responses in the brain. Early studies showed a time- and dose-dependent increase in mRNA levels of muscarinic receptors by ET-1 in cultured cerebellar granule cells [49]. Moreover, vagal activation was observed after ET-1 administration either intracisternally [50] or selectively in the dorsal vagal complex [51] of anesthetized rats. Lastly, bradycardia was observed after intrathecal injection of ET-1 at high dosages in conscious rats, often leading to bradycardic arrest [43].

The effects of ET-1 on vagal activity appear to vary, depending on the type and intensity of AES. Important data on this topic come from the work by Kurihara et al. [38], who examined the role of ET-1 in vagal responses after AES in wild-type and heterozygous ET-1-knockout mice (having low plasma ET-1 levels). The latter animal cohort exhibited diminished autonomic responses to intruder stress but responded to restraint more intensely than wild-type mice. Moreover, in a clinical study on patients with chronic stable coronary artery disease, vagal withdrawal (assessed by HRV) after anger recall correlated with plasma ET-1 levels [52]. Thus, further research is warranted on the effects of ET-1 during various types of AES.

### 4.6. Rhythm Disturbances in Wild-Type and ET_B_-Deficient Rats

In keeping with previous findings [8], we observed PVCs in wild-type rats during AES. Interestingly, frequent PVCs were also observed in this strain during recovery, coinciding with prolonged vagal withdrawal and enhanced voluntary motion. This finding indicates prolonged anxiety after AES and underscores the need to encompass long observational periods in rat models of AES. Extended observation increases a model’s translational value, based on the delayed ventricular tachyarrhythmias frequently reported in clinical and epidemiologic studies of AES [5,6,7,9,10].

Contrasting the rhythm disturbances in wild-type rats, we observed bradyarrhythmic events, primarily in the form of sinus pauses, in ET_B_-deficient rats; the number of these episodes increased markedly during AES, with a subsequent decrease during recovery. This (rather unexpected) finding has been rarely reported in rat models of AES; hence, its relevance to human pathophysiology is uncertain, although it resembles vagal stimulation and bradycardia during freezing reactions after fearful emotions [42].

### 4.7. Freezing Reactions to Fear

The features observed in ET_B_-deficient rats may be considered representative of a freezing reaction, a view supported by the low voluntary activity post-AES in these animals. Indeed, freezing reactions in animal models are characterized by a motionless posture after a threat of moderate intensity causing fear [53]. Such complex responses are likely accompanied by accentuated sympatho-vagal interaction, leading to vagal dominance, “fear bradycardia” and syncope [42].

### 4.8. Neurocardiogenic Syncope

Neurocardiogenic syncope consists of excessive vagal stimulation leading to bradycardia in response to various stimuli, including AES. The link between high baseline ET-1 levels and neurocardiogenic syncope was suggested in small series of pediatric [25] and adult [54] patients, with further evidence provided by gene studies [55]. Interestingly, such responses are clinically observed invariably in certain personality traits with depressive characteristics [56] that have been linked to high ET-1 levels [57]. Notably, the onset of bradycardia in our recordings (Figure 10) resembles the responses observed during the provocation of neurocardiogenic syncope by head-up tilt testing in clinical practice. We feel that the intriguing hypothesis of ET-1 mediating vagal responses in neurocardiogenic syncope merits further study.

### 4.9. Strengths and Limitations

We examined the effects of ET-1 on AES in conscious rats. We utilized a well-characterized rat model with chronically elevated ET-1 levels [28], thereby circumventing the disadvantages associated with chronic exogenous ET-1 administration. However, it should be noted that ET-1 levels in ET_B_-deficient rats were not confirmed in our experimental animal cohort. Another strength of our study is the assessment of sympathetic and vagal responses, as well as rhythm disturbances, over a prolonged observational period, thereby providing information on a clinically important timeframe. In addition to ventricular tachyarrhythmias resulting from sympathetic activation, our findings draw attention to bradyarrhythmic events as important rhythm disturbances in response to AES.

Despite these merits, three limitations should be acknowledged: First, our experiments included only one AES protocol, regarded as specific for investigating fear. However, rats respond differently to various stressors, such as social defeat, as discussed above; hence, our results do not apply to other common conditions, such as anger or grief. Second, our experiments were performed in the setting of a normal myocardium, even though AES can trigger acute coronary syndromes. Further research is warranted on the substantially different autonomic responses and arrhythmogenesis under such circumstances. Third, our study protocol did not include histologic analysis of heart or brain specimens.

## 5. Conclusions

Sympathetic activation, prolonged vagal withdrawal and frequent PVCs occur in response to AES in rats under conditions of a normal myocardium. ET_B_-deficient rats, a strain with previously demonstrated high plasma ET-1 levels, display markedly blunted responses to AES. Although both autonomic arms are affected in this rat strain, sustained low sympathetic activity results in vagal dominance mainly during recovery, associated with low voluntary activity and bradycardia. Our findings strengthen the link between ET-1 and autonomic responses to AES and provide further insights into the pathophysiology of stress-induced tachy- and bradyarrhythmias. Whether AES elicits more complex autonomic responses or rhythm disturbances in other settings remains to be investigated.

## Figures and Tables

**Table 1:** Baseline characteristics.

Variables		Wild-Type	ET_B_-Deficient	*p* Value *
Sympatho-vagal balance	Mean HR (bpm)	314 ± 53	337 ± 34	0.1029
	LF/HF **	0.021 ± 0.011	0.011 ± 0.0007	**0.0005**
	SDNN ** (ms)	4.122 ± 4.638	5.309 ± 4.144	0.3984
	RMSSD ** (ms)	7.285 ± 8.242	9.562 ± 7.616	0.3700
Sympathetic activity	Power LF	1.874 ± 0.955	1.129 ± 0.0697	**0.0012**
	SNSi **	451 ± 212	325 ± 75	**0.0172**
Vagal activity	Power HF	97.82 ± 1.02	98.66 ± 0.08	**0.0007**
	PNSi **	−5.459 ± 0.179	−5.547 ± 0.1056	0.0671
Bradyarrhythmias	Sinus pauses	0	0.50 ± 1.00	**0.0197**
	AV ** block episodes	0	0	(−)
Tachyarrhythmias	PVCs **/h	0.250 ± 0.55	0.35 ± 0.87	0.8540
	Couplets/h	0	0.10 ± 0.44	0.3421
	Triplets/h	0	0.15 ± 0.36	0.0803
Voluntary activity	Activity (counts/h)	444 ± 572	477 ± 428	0.8346

* Significant values are printed in bold. ** Abbreviations: LF: low frequency, HF: high frequency, SDNN: standard deviation of inter-beat intervals, RMSSD: root mean square of the successive differences, SNSi: sympathetic nervous system index, PNSi: parasympathetic nervous system index, AV: atrioventricular, PVCs: premature ventricular contractions.

## Data Availability

Data supporting the reported results can be found after contacting E-T.M. by email (elenimouch@gmail.com).

## Supplementary Materials

The following supporting information can be downloaded at: https://www.mdpi.com/article/10.3390/biology12111401/s1, Table S1: List of abbreviations.
