# Supplementary material for: Endothelin Modulates Rhythm Disturbances and Autonomic Responses to Acute Emotional Stress in Rats
Source: Biology (Basel). 2023 Nov 5;12(11):1401. doi: 10.3390/biology12111401 (PMC10669295; doi:10.3390/biology12111401)
Supplement: Supplementary file 1 [file biology-12-01401-s001.zip › biology-2641122-supplementary.pdf]

Table S1: List of abbreviations

Abbreviation	Explanation
AES	acute emotional stress
AJS	air jet stress
AV	atrioventricular
BP	blood pressure
ECG	Electrocardiogram
ET	endothelin
ET_A- receptors	endothelin (A) receptors
ET_B-receptors	endothelin (B) receptors
ET_B-deficient rats	rescued phenotype of rats carrying a natural deletion in the gene encoding for the endothelin-B (ET_B) receptor
HF	high frequency
HR	heart rate
HRV	heart rate variability
PVC	premature ventricular contraction
PNSi	parasympathetic (vagal) nervous system index
RMSSD	root mean square of the successive differences of inter-beat intervals
SDNN	standard deviation of inter-beat intervals
SNSi	sympathetic nervous system index
w/t	wild type
